# Physician Attitudes towards Pharmacological Cognitive Enhancement: Safety Concerns Are Paramount

**DOI:** 10.1371/journal.pone.0014322

**Published:** 2010-12-14

**Authors:** Opeyemi C. Banjo, Roland Nadler, Peter B. Reiner

**Affiliations:** National Core for Neuroethics, University of British Columbia, Vancouver, British Columbia, Canada; Charité-Universitätsmedizin Berlin, Germany

## Abstract

The ethical dimensions of pharmacological cognitive enhancement have been widely discussed in academic circles and the popular media, but missing from the conversation have been the perspectives of physicians - key decision makers in the adoption of new technologies into medical practice. We queried primary care physicians in major urban centers in Canada and the United States with the aim of understanding their attitudes towards cognitive enhancement. Our primary hypothesis was that physicians would be more comfortable prescribing cognitive enhancers to older patients than to young adults. Physicians were presented with a *hypothetical* pharmaceutical cognitive enhancer that had been approved by the regulatory authorities for use in healthy adults, and was characterized as being safe, effective, and without significant adverse side effects. Respondents overwhelmingly reported increasing comfort with prescribing cognitive enhancers as the patient age increased from 25 to 65. When asked about their comfort with prescribing extant drugs that might be considered enhancements (sildenafil, modafinil, and methylphenidate) or our hypothetical cognitive enhancer to a normal, healthy 40 year old, physicians were more comfortable prescribing sildenafil than any of the other three agents. When queried as to the reasons they answered as they did, the most prominent concerns physicians expressed were issues of safety that were not offset by the benefit afforded the individual, even in the face of explicit safety claims. Moreover, many physicians indicated that they viewed safety claims with considerable skepticism. It has become routine for safety to be raised and summarily dismissed as an issue in the debate over pharmacological cognitive enhancement; the observation that physicians were so skeptical in the face of explicit safety claims suggests that such a conclusion may be premature. Thus, physician attitudes suggest that greater weight be placed upon the balance between safety and benefit in consideration of pharmacological cognitive enhancement.

## Introduction

Public acceptance of new technology ranges from wholehearted embrace to outright rejection of radical technological change [1]–[3]. Few advances bring this divergence of opinion into such stark relief as the subject of cognitive enhancement in healthy persons, in part because of the value society places upon cognitive ability [4], [5]. With technology increasingly permeating every corner of modern life, it comes as no surprise that pharmacological approaches which might ameliorate the normal cognitive decline that accompanies aging and even enhance cognitive function in young adults have garnered much interest.

To date, discussion regarding the propriety of pharmacological cognitive enhancement has primarily been the domain of bioethicists, philosophers, and scientists, with journalists and enthusiastic consumers joining the fray at regular intervals [6]–[27]. In contrast, the views of physicians on this subject have received scant attention [28]. This is not to say that the subject of the challenges that cognitive enhancement brings to the clinic has gone unconsidered, but rather that the issue has been largely restricted to thought leaders in academic medicine [10], [18], [29]–[31]. Given their roles as key decision makers in the adoption of new technologies into medical practice, and moreover as individuals likely to be called upon as the gatekeepers in dispensing pharmaceutical cognitive enhancers, an examination of physician attitudes on this topic struck us as overdue.

It may be instructive at the outset to draw attention to two different conceptions of pharmacological cognitive enhancement. The first recognizes that intellectual acuity declines as humans age, even in the absence of frank disease. Distinct from the prodromic cognitive decline that precedes dementia [32], the cognitive decline that accompanies normal aging – formally termed age-associated memory impairment or AAMI – is disturbing to many [33]. The prevalence of AAMI, ranging from 38%–58% for normal adults in their 60′s [34], [35], identifies the phenomenon as a defining feature of normal aging, and situates AAMI at the indistinct interface of normalcy and pathology. Moreover, because the cognitive decline of AAMI is a *decline*, one can readily imagine many to be sympathetic to the notion that in this instance, pharmacological tools might constitute *restoration*.

The second conception is one that focuses much more directly upon *enhancing* human traits, and is exemplified by discussions regarding pharmacologically enhancing cognition in young adults who exhibit no measurable cognitive decline. It is here that the full range of opinions on the topic emerges, with attitudes ranging from enthusiasm and moral praiseworthiness through reasoned skepticism and even overt antagonism [10], [14], [17], [21], [24], [36], [37]. Intuitions regarding the moral propriety of enhancement and restoration are themes that recur, often implicitly but nearly invariably, in discussions of cognitive enhancement in medical practice.

Our primary objective was to examine physician views towards prescribing pharmacological cognitive enhancers to cognitively normal individuals. However, even asking the question raises a larger issue of concern to many physicians: to what extent is it appropriate to use modern medical technology to enhance the healthy? In recent years, physicians have increasingly been asked to prescribe drugs that fall in the ‘grey zone’ between treatment and enhancement [27], [38]–[40], but the process has been more haphazard than deliberate: there has been no systematic program by which the medical community has come together to decide what avenues of treatment are appropriate. Rather, responsibility for these important decisions has been left in the hands of the pharmaceutical industry and the regulatory authorities; some have suggested that the results have been less than ideal [27], [38], [41]–[43]. Thus, one objective of our study was to begin to provide an opportunity for physicians, in particular general practitioners who are most likely to be asked to prescribe such drugs in the future [44], to express their attitudes towards enhancement in general, and cognitive enhancement in particular.

Irrespective of whether physicians are explicitly aware of the nosology of age-associated memory impairment, they are implicitly aware of the fact that there exists a normal decline in cognitive function in older individuals which is distinct from that seen in dementia. Recognizing this, we reasoned that physicians would feel that helping older patients overcome cognitive decline is more akin to restoration than enhancement, and therefore is better aligned with the proper goals of medicine than treating younger patients who do not experience such decline and would be perceived as pursuing enhancement rather than restoration. This reasoning led us to our primary hypothesis that physicians would be more comfortable prescribing cognitive enhancers to healthy older patients than to healthy young adults. We also reasoned that familiarity was an important consideration for physician prescribing behavior, and as a result hypothesized that physicians would also feel more comfortable prescribing existing drugs that are sometimes considered enhancers as compared to a hypothetical drug specifically designed and marketed as a cognitive enhancer. Here, we present data from over 200 physicians from across the United States and Canada who responded to our survey.

## Materials and Methods

### Ethics Statement

The study was approved by the University of British Columbia Behavioral Research Ethics Board (H09-00340).

We recruited primary care physicians practicing in major urban centers in Canada and the United States by mailing out letters to addresses in publicly available databases and by posting free advertisements in medical association newsletters. Both the letter and the newsletter ad highlighted the goals of the study and directed interested participants to a web-based survey. The incentive for participating was primarily to enable physicians express their opinions on a conversation that had largely excluded them, however, participants from each country also had the option of entering into a random draw for their choice of an 8 GB Apple iPod touch®, or a $250 gift card to the bookstore of their choice upon completion of the survey. The monetary incentive was deliberately kept minimal in order to ensure that physicians had sufficient interest in the research subject and thereby result in a more robust data set.

The introductory paragraph of the survey briefly reviewed normal age-related cognitive decline in healthy individuals and introduced physicians to a *hypothetical* pharmaceutical agent that had been approved by the regulatory authorities as a cognitive enhancer for use in healthy adults, and was characterized as being safe, effective, and without any significant adverse side effects. The paragraph also reminded the physicians that the cognitive decline associated with aging is *not* a disease, and that objective measures of such normal cognitive decline can begin to appear as early as the late 30′s. Demographic data included respondents' professional background, age, sex, ethnicity, place of birth, and primary residence.

The survey began by probing physicians' familiarity with cognitive enhancement in healthy persons, and then progressed to assess physician attitudes towards patients' cognitive health. We asked whether or not physicians probed cognitive function as a part of routine physical exams in patients in three different age groups; 25–40, 41–59, and 60 and older, and asked them to indicate their reasons for probing cognitive health from lists we provided. Next, we asked physicians to rate how comfortable they felt prescribing the hypothetical cognitive enhancer to three different patients: a 25-year-old, a 45-year-old, and a 65-year-old, all of whom were otherwise healthy, but had come reporting symptoms of age-related cognitive dysfunction. In order to assess the impact of patients giving reasons for requesting the drug upon physician attitudes, we again presented three patients: a 25-year-old graduate student seeking to cope with the stress of graduate school, a 45-year-old employee hoping to improve productivity, and a 65-year-old individual feeling concerns about his ability to perform everyday activities. Lastly, we probed physicians' attitudes towards prescribing our hypothetical cognitive enhancer and three other pharmaceutical agents sometimes considered enhancers – sildenafil, modafinil, and methylphenidate – in a 40-year-old reporting symptoms consistent with the label indications for each respective drug. We included modafinil and methylphenidate because these agents are those that are most often mentioned in the cognitive enhancement literature [7]–[25], and included sildenafil because it satisfies the criteria for an enhancer but acts on the body rather than the brain. Responses to all rating questions were made on a 7-point Likert scale, with anchors at 1 (less comfortable) and 7 (more comfortable). The questions provided the respondents with the opportunity to select the reasons influencing their decisions, as well as the opportunity to freely respond with comments.

Upon completing the survey, participants were given the opportunity to leave their email addresses either to be re-contacted for their willingness to participate in a future study, and/or to be notified of the study results when it became available, and/or to enter into the random prize draw. Respondents also had the option not to select any of the options and simply submit the survey. All respondents gave consent to participate, and to the use of the data they provided.

The survey was hosted on the online survey tool Zoomerang©, and was kept open for a period of three months. Quantitative data was analyzed using the GraphPad Prism 3.0 software (GraphPad Software Inc., San Diego, CA, U.S.A). Student's t-tests and one-way ANOVAS were used to assess statistical significance in differences between groups; data was considered statistically significant when P values were less than or equal to 0.05. Content analysis for the open-ended responses was manually performed using the conceptual analysis method: coding was performed in an interactive manner in which concepts were not predefined, but rather were developed during the coding process as new themes were identified [45]. Concepts were coded based on the frequency of occurrence, and themes with greater emphasis were identified based on the number of times they appeared in the comments. To determine inter-coder agreement, 15% of the open-ended responses were randomly selected and assigned to a second coder who was not involved with the initial coding process. Inter-coder percent agreement was 95%, and Cohen's Kappa coefficient (κ) was 0.54 (ReCal Software) [46].

## Results

A total of 212 physicians responded to the survey (148 residing and practicing in the USA, 64 residing and practicing in Canada); 88% were general practitioners. The demographic data for respondents is shown in Table 1. Because some physicians were recruited using advertisements placed in newsletters, we are unable to provide corresponding demographic data on physicians in the catchment areas, nor an accurate response rate. Using the data from physicians who were contacted via letter in Vancouver and Toronto as a guide, we estimate the response rate as ∼4%. Thus, the responding physicians should be viewed as comprising a *convenience sample* of physicians rather than a *representative sample*. Sixty one percent (61%) of respondents indicated that they had read articles in either the popular press or the scientific literature on the subject of cognitive enhancement within the last 5 years. Four percent (4%) of respondents indicated they were “very familiar” with the subject; 57% rated themselves as being “not familiar” with the subject, while 39% indicated they were “somewhat familiar” with the subject.

**Table 1:** Physician Demographic Data.

Specialty	General Practitioners; 88%Other; 12%
Sex	Males; 55%Females; 45%
Age	25–40; 36%41–59; 45%60+; 19%
Number of years in practice	1–10; 45%11–20; 24%20+; 31%

The key demographic information collected from all survey participants.

Next, we probed physician attitudes to cognitive health in patients in three different age groups: 25–40, 41–59, and 60 and above. Eighty-four percent (84%) of physicians did not routinely probe cognitive function in patients aged 25–40, and 65% of physicians did not routinely probe cognitive function in patients aged 41–59 (Figure S1); in both instances, the primary reason they selected for not probing was that the patient was neither showing nor complaining of cognitive deficits (Table S2). When asked to freely respond, most physicians indicated that their practice was “treatment-focused” hence they didn't probe cognitive function in these age groups. However, by the time patients were aged 60 and above, 79% of the physicians surveyed indicated that they routinely probed cognitive function. The primary reason they selected from the list we provided was age-appropriateness; most physicians also indicated in their free response that they often probed cognitive functions in this age group to assess for early stages of memory loss.

**Figure 1:**
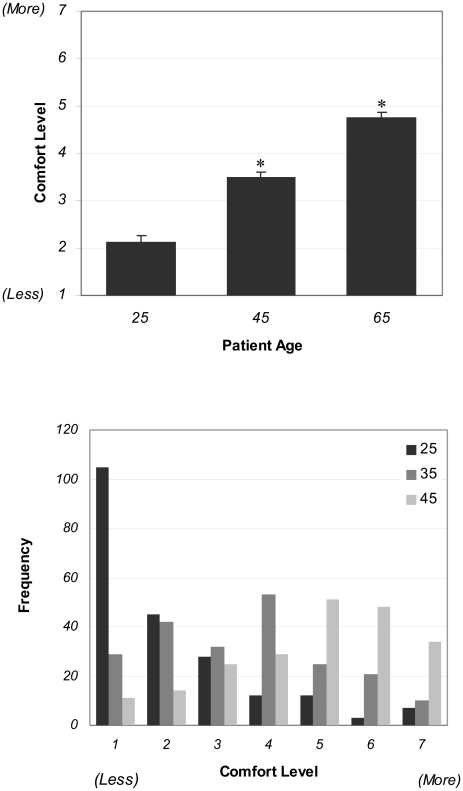
Physicians' Comfort Rating with Prescribing Cognitive Enhancers to Patients of Differing Ages. (A) Mean physician comfort rating with prescribing the hypothetical cognitive enhancer to patients of differing ages. Physicians reported increasing comfort with prescribing cognitive enhancers as the patient's age increased from 25 to 65 (P<0.001). (B) Frequency of occurrence of each response on a 7-point Likert scale, with anchors at 1 (less comfortable) and 7(more comfortable).

The next set of questions directly addressed our primary hypothesis that physicians would be more comfortable prescribing cognitive enhancers to older patients than to young adults. We queried how comfortable physicians would feel prescribing a hypothetical cognitive enhancer to individuals who were 25, 45, or 65 years of age. Respondents overwhelmingly reported increasing comfort with prescribing cognitive enhancers as the patient age increased from 25 to 65, and the differences between age groups were statistically significant (P<0.001; Figure 1). We performed further stratified data analysis to assess whether or not physician age, sex, or their self-reported familiarity with cognitive enhancers correlated with their comfort with prescribing to patients in the 3 different age groups (Figure S3). We found no significant differences between the groups based on physician age or familiarity with cognitive enhancers; however, we did find that while both male and female physicians were similarly uncomfortable with prescribing cognitive enhancers to the 25-year-old patient, male physicians rated themselves as being significantly more comfortable prescribing cognitive enhancers to both the 45- and the 65-year-old patients.

We also assessed whether or not physicians would be swayed if the patients gave reasons for requesting the cognitive enhancer rather than simply reporting symptoms of cognitive dysfunction. The same 3 patients as before were presented again, only now the 25-year-old was a graduate student who was looking to cope with the stress of graduate school; the 45-year-old, a worker looking to improve work productivity; and the 65 year old an older individual having concerns about his ability to perform everyday activities. The resultant data were not significantly different than their responses without reasons (P>0.05).

The survey provided the respondents with a list of possible reasons as to why they may have rated their comfort levels with prescribing to patients in different age groups as they did. The reasons and their responses are shown in Table 2.

**Table 2:** Reasons Affecting Physician Comfort with Prescribing Cognitive Enhancers to Patients of Different Ages.

*Reasons*	*25*	*45*	*65*
Fear of misuse	125	93	24
Patient does not need the drug	116	93	32
Availability of non-pharmacological methods of achieving the same goals	93	84	53
Undermines the values of personal effort	48	42	10
To improve patient's overall health and wellness	38	69	134
Fear of legal liability	38	33	16
To help patient succeed	35	51	64
To improve daily living	25	74	146
It constitutes a form of cheating	26	13	3
Your cultural values	19	17	15
Respect for patient's autonomy	15	34	60
Drug is age-appropriate	13	38	109
Patient's socio-economic status	7	9	14
Your religious beliefs	4	1	3

Table 2 shows the total number of physicians who selected individual reasons from the list we offered as to why they rated their comfort levels as they did. Respondents were able to select as many of the reasons as they felt was applicable.

While the predominant reasons for feeling uncomfortable prescribing to the younger patient were a) that the patient did not need the drug and b) fear of misuse, these sentiments were markedly diminished in the case of the older patient, to whom most physicians felt more comfortable prescribing in order to help improve daily living and overall health and wellness.

Recognizing that the list of reasons we gave physicians may not be exhaustive, we also provided the opportunity for additional comments. In the free responses (Table S1), most physicians expressed safety concerns about the drug even though we had clearly indicated that the drug was safe, approved by regulatory authorities, and devoid of significant side effects. Although safety concerns remained predominant for all three patients, fewer physicians expressed these concerns as the patient's age increased from 25 to 65 (37% to 28%). Rather, more physicians expressed empathy and a desire to help the 65-year-old patient maintain a good quality of life. Respondents also commented on the drug being perceived as an unnecessary medical intervention, particularly in the younger patient; concerns about enhancement falling beyond the scope of medicine's proper roles also emerged as prevalent for scenarios involving patients in all age groups.

**Figure 2:**
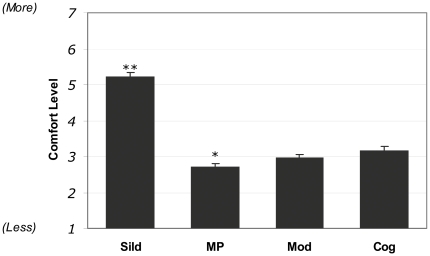
Physicians' Comfort Rating with Prescribing Sildenafil, Methylphenidate, Modafinil, and a Hypothetical Cognitive Enhancer. Physicians reported being significantly more comfortable prescribing sildenafil compared to the other 3 drugs (P<0.001); while methylphenidate was rated significantly lower (P<0.01) when compared with sildenafil and the cognitive enhancer, but not modafinil.

In order to assess how physicians feel about prescribing cognitive enhancers in comparison with other drugs that are already commonly prescribed but are sometimes considered enhancers, we asked physicians to rate how comfortable they felt prescribing any of the following: the hypothetical cognitive enhancer, sildenafil, modafinil, and methylphenidate. In each instance, the patient was a 40-year-old reporting symptoms consistent with the label indications for the respective drug. Physicians indicated they were significantly more comfortable prescribing sildenafil as compared to the other 3 drugs, all of which they reported being quite uncomfortable prescribing (Figure 2). As with prescribing cognitive enhancers to patients of differing ages, we also found that male physicians rated themselves as being significantly more comfortable than the females with prescribing sildenafil, modafinil, and the hypothetical cognitive enhancer to the 40-year-old patient (Figure S4).

To further assess physician angst about safety, as well as to clarify why the majority of respondents were significantly more comfortable prescribing sildenafil relative to the three other drugs, we performed a brief follow-up survey specifically to address those two issues. We queried all of the 66% of the initial survey participants who had expressed their willingness to be re-contacted for a future study, asking them how comfortable they were prescribing the hypothetical cognitive enhancer to a 25-year-old, this time encouraging them to specifically assume the availability of favourable long-term safety data (Table S3). A subsequent question shared our data from Figure 2 on sildenafil and the three other drugs, and asked physicians to comment as to why they may have responded as they did. The data revealed that the availability of long-term safety data did indeed convince some physicians, resulting in a statistically significant (P<0.001) increase in the average comfort rating compared to the previously described 25 year old patients. Nonetheless, physicians remained clearly in the uncomfortable range of the scale (mean  = 3.3) (Figure S2).

When asked to freely respond on their answer choices, physicians' overarching concerns remained safety issues that were not necessarily offset by the benefits to the patient. A number of physicians also expressed discomfort with the idea of cognitive enhancement in and of itself, raised concerns about fairness, and also reiterated that they felt as though enhancement moves beyond the proper goals of medicine.

When asked why they rated themselves as being more comfortable with prescribing sildenafil than the three other drugs, most physicians indicated an increased familiarity with the drug, and a better safety profile (Table S4). Other concerns included the abuse potential of stimulant drugs, the ability to objectively measure the “success” of the drug, and the inclination to be more cautious when prescribing any drug that affects brain function. In their overall comments on their views on cognitive enhancement, physicians continued to state safety worries as the primary issue of concern (Table 3). The overall comments demonstrated that most physicians were intensely risk-averse, and had a high distrust for pharmaceuticals, particularly when the drug intervention is for enhancement purposes.

**Table 3:** Physician General Comments on Prescribing Cognitive Enhancers.

*Themes*	*Percentage of Comments*
Safety concerns	49%
Unnecessary medical intervention	15%
Lack of familiarity with subject	15%
Availability of Non-pharmacological Alternatives	12%
Efficacy concerns	9%
Empathy for patient/To help maintain quality of life	9%
Age-appropriateness	6%
Distributive Justice	5%
Treatment-focused physician	5%
Disease mongering	5%
Dependent on patient's history	3%
Cost	3%
Respect for patient's autonomy	2%
Coercion	1%

At the end of the survey, physicians received an optional comment box to provide any additional views they may have about prescribing cognitive enhancers. 59% of the total respondents left comments; these comments were grouped into themes using the conceptual analysis method.

The final question in the survey asked about physicians' personal use of enhancers. Over 75% of the respondents stated they routinely drank caffeinated products, with their primary reasons being for mental alertness, and taste. When asked if they would personally take a cognitive enhancer (of proven efficacy, bearing regulatory approval, and devoid of significant side effects), only 29% of the respondents answered with a definitive “no”, 23% of the respondents stated “yes,” while 48% stated “maybe.”

## Discussion

Our primary hypothesis was that physicians would feel more comfortable prescribing cognitive enhancers to older patients than to young adults, notwithstanding the fact that all of the patients presented to them were normal, healthy adults. Physicians overwhelmingly endorsed this view, reporting increasing comfort as patient age increased from 25 to 65; this finding was similar whether or not patients provided lifestyle reasons to support their requests for the drug. When presented with a predefined checklist which offered some possible reasons that physicians might offer in support of their attitudes towards prescribing pharmacological cognitive enhancers, physicians identified improvements in patient quality of life as a major factor in motivating them to prescribe these drugs to 65 year olds, while they characterized concerns about misuse and the absence of a true need of the drug as staying their hand in writing prescriptions to 25 year olds.

One reasonable interpretation of these data is that they reveal differing attitudes towards *enhancement* as opposed to *restoration*: when considering younger adults, physicians viewed cognitive enhancers as an unnecessary enhancement, but when evaluating older adults who may be experiencing the normal cognitive decline associated with aging, physicians viewed the treatment as restorative. Thus physicians, whether through scholarship, implicit reasoning, or other forms of knowledge, are generally attuned to the tensions that accompany discussions of the treatment-enhancement distinction in medical practice [38] The physicians in our data set generally expressed sentiments which endorsed a relatively conservative view of the enhancement debate: few physicians expressed enthusiasm about the opportunity to use modern technology to produce humans whose capabilities exceeded what is general considered normal. Moreover, arguments grounded in physicians' conception of the proper role or purview of medical practice emerged as a prevalent theme in our content analysis of free responses. Whether it is the case that skepticism of the enterprise of enhancement drives this view of medicine or vice versa is difficult to speculate; also unclear is whether classifying *cognitive* enhancement as beyond medicine's scope prompts physicians to reconsider the status of other, perhaps more commonly accepted, interventions that we may reasonably term enhancements.

An unexpected outcome of our study was the degree to which physicians mistrust safety claims regarding pharmaceuticals. When allowed to freely comment on their views on prescribing cognitive enhancers, 49% of physicians who responded to this optional question expressed safety concerns as dominating their rumination on the topic. Most notable was the observation that these attitudes persisted even though many of the physicians acknowledged that they understood that the hypothetical cognitive enhancer was approved by the regulatory authorities and had been described as devoid of any significant side effects (for a sample of physician comments, see supplementary data). So striking were these findings that we carried out a follow-up survey to the subgroup of physicians who agreed to be re-contacted, explicitly stating in the follow-up question that respondents should assume that all safety concerns have been put to rest by convincing long-term data. While this further clarification increased the comfort level of physicians with prescribing cognitive enhancers, the average comfort rating merely went up from 2.3 to 3.3 on a scale of 1–7 (with the anchors of 1 and 7 indicating less and more comfort with prescribing, respectively), indicating that even under these conditions physicians viewed even data-backed safety claims as unconvincing.

The second hypothesis that we tested was that physicians would feel more comfortable prescribing extant drugs that are sometimes considered enhancers as compared to the hypothetical cognitive enhancer presented in our scenario. When physician attitudes towards prescribing sildenafil, modafinil, methylphenidate, or the hypothetical cognitive enhancer to a healthy 40-year-old were probed, the results indicated that the physicians were only comfortable prescribing sildenafil, suggesting that the hypothetical nature of our cognitive enhancer could not fully account for physician concerns in the earlier part of the study.

We provided physicians with an opportunity to freely comment on this finding in our follow-up study, asking them why *they* thought they might have responded as they did. The primary reason physicians gave for this pattern of responses was greater familiarity with sildenafil. This observation reinforces the overall tenor of responses by physicians in the first part of this study, namely that safety issues with drugs that are viewed as enhancers dominate their list of concerns, and that these can only be mitigated by the long-term success of the agent in daily practice.

Taken together, our data suggest that physicians are keenly aware of the ethical landscape involved in prescribing cognitive enhancers. Moreover, they appear to use this information in appraising the tradeoff between safety and benefit when making decisions about the propriety of prescribing such drugs for *enhancement* as distinguished from *restoration*. At the same time, physicians overwhelmingly utilized arguments from the perspective of safety to help them rationalize their decisions regarding prescribing cognitive enhancers.

The issue of safety is often raised and summarily dismissed in the debate over pharmacological cognitive enhancement by deferring to the authority of regulatory approval [21] The observation that physicians remained skeptical in the face of explicit safety claims suggests that such a conclusion may be premature. These data lend empirical force to the notion that regulatory authorities would be well advised to maintain the highest standards possible with respect to safety claims when evaluating pharmaceutical agents that may be construed as being enhancements. Finally, the findings of this study more generally forewarn that as pharmacological cognitive enhancement moves from discourse to reality, it will increasingly be important to move the debate beyond academic analysis to include objective engagement of individuals who are most likely to be affected.

## Supporting Information

Figure S1 Physician Attitudes to Patients' Cognitive Health. Over 80% of physicians reported not routinely probing cognitive function in patients aged 25-40, and 65% of physicians also did not routinely probe cognitive function in patients' aged 41-59. However, 79% of the physicians surveyed routinely probe cognitive function in patients' aged 60 and above.
(1.03 MB TIF)

Figure S2 Physicians' Comfort Rating with Prescribing Cognitive Enhancers to 25-year-old Patients in Different Scenarios. Figure S2 compares how physicians rated their comfort levels with prescribing the described cognitive enhancer to three 25-year-old patients in 3 different scenarios: one who came in simply reporting symptoms of cognitive dysfunction (no reason); the graduate student (with reason); and the patient presented in the re-contact survey, with all safety concerns presented as having been laid to rest. The data revealed a significant increase (P<0.001) in comfort rating after safety concerns were laid to rest, although the mean rating was still 3.275.
(1.14 MB TIF)

Figure S3 Stratified Analysis of Physicians' Comfort Rating with Prescribing Cognitive Enhancers to Patients. Mean physician comfort rating with prescribing the hypothetical cognitive enhancer to patients of differing ages stratified by physician age, sex, and familiarity with cognitive enhancers. (A) There was no significant difference between physicians in different age groups (25–40; 41–59; 60+), P>0.05. (B) Male physicians were significantly more comfortable with prescribing the cognitive enhancer to 45- and 65-year-old patients (P<0.05) compared to the female phyicians. (C) There was no significant difference (P>0.05) in comfort level between physicians who rated themselves as being “familiar” or “unfamiliar” with cognitive enhancement in healthy persons.
(2.42 MB TIF)

Figure S4 Male and Female Physicians' Comfort Rating with Prescribing Sildenafil, Methylphenidate, Modafinil, and the Cognitive Enhancer. Male physicians reported being significantly more comfortable prescribing sildenafil (P<0.05); modafinil (P<0.005); and the hypothetical cognitive enhancer (P<0.005), when compared with female physicians.
(1.16 MB TIF)

Table S1 Selected Comments on Physician Views on Prescribing Cognitive Enhancers.
(0.03 MB DOC)

Table S2 Physician Reasons for Probing or not Probing Cognitive Health in Patients. Table S2 shows the percentage of physicians that selected individual reasons from the list we offered as to why they probe or do not probe cognitive health in patients of different age groups during routine visits. Respondents were able to select as many of the reasons as they felt was applicable.
(0.04 MB DOC)

Table S3 Comments on Prescribing Cognitive Enhancers to a 25-year-old, Assuming Long-term Favorable Safety Data. Physicians were asked to freely respond on the question of prescribing the hypothetical cognitive enhancer to a 25-year-old patient assuming all the safety concerns they previously had have been laid to rest with long-term convincing data. Their comments were grouped into themes using the conceptual analysis method.
(0.03 MB DOC)

Table S4 Physician Comments on being more Comfortable Prescribing Sildenafil Compared to the Other Three Drugs. Physicians were asked to freely respond on why they feel the data showed that the majority of the respondents were significantly more comfortable prescribing sildenafil compared to the other 3 drugs. Their comments were grouped into themes using the conceptual analysis method.
(0.03 MB DOC)
