# Supplementary figures and images for: Physician Attitudes towards Pharmacological Cognitive Enhancement: Safety Concerns Are Paramount
Source: PLoS One. 2010 Dec 14;5(12):e14322. doi: 10.1371/journal.pone.0014322 (PMC3001858; doi:10.1371/journal.pone.0014322)

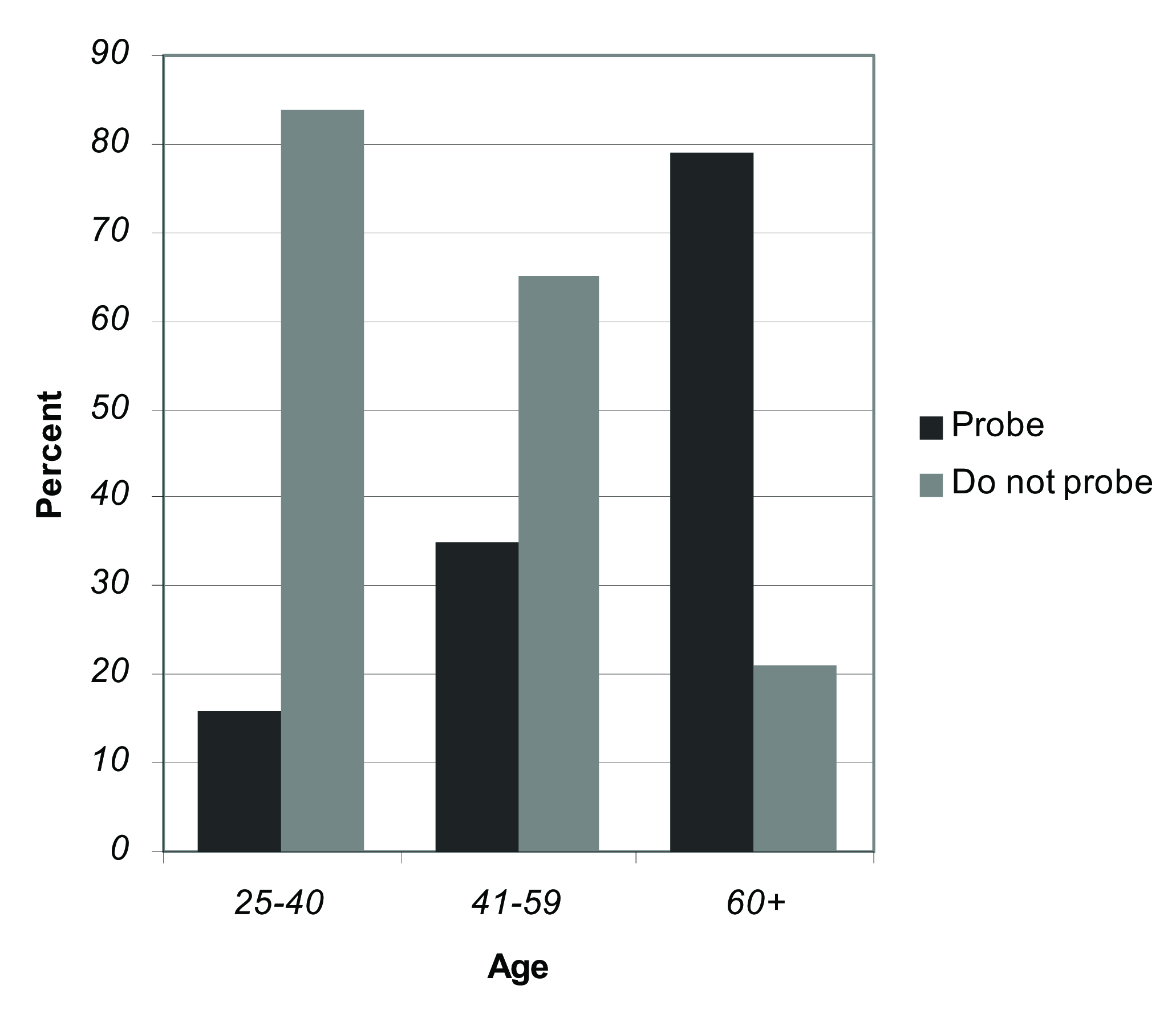

Supplement: Figure S1 — Physician Attitudes to Patients' Cognitive Health. Over 80% of physicians reported not routinely probing cognitive function in patients aged 25-40, and 65% of physicians also did not routinely probe cognitive function in patients' aged 41-59. However, 79% of the physicians surveyed routinely probe cognitive function in patients' aged 60 and above. (1.03 MB TIF) [file pone.0014322.s001.tif]

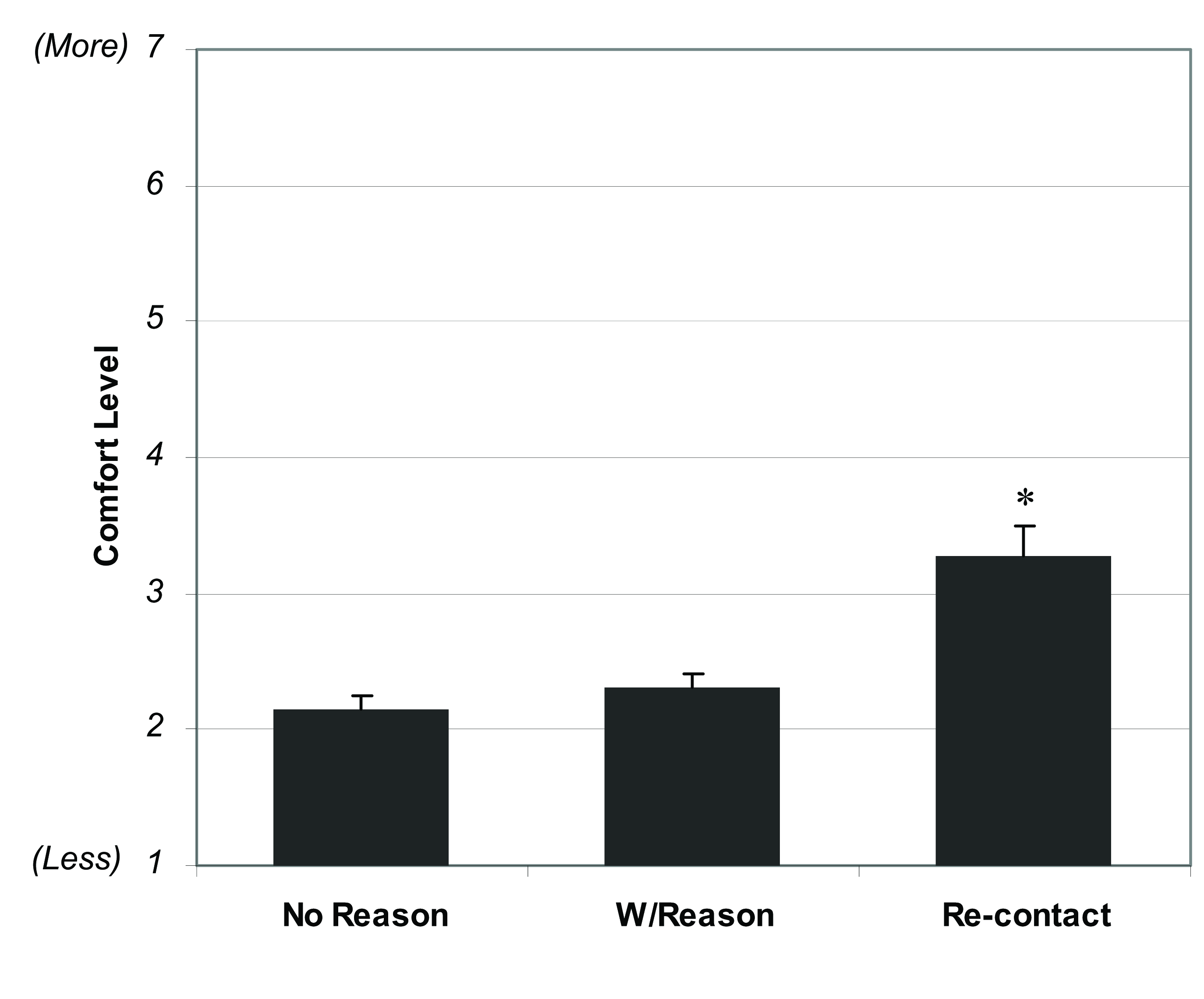

Supplement: Figure S2 — Physicians' Comfort Rating with Prescribing Cognitive Enhancers to 25-year-old Patients in Different Scenarios. Figure S2 compares how physicians rated their comfort levels with prescribing the described cognitive enhancer to three 25-year-old patients in 3 different scenarios: one who came in simply reporting symptoms of cognitive dysfunction (no reason); the graduate student (with reason); and the patient presented in the re-contact survey, with all safety concerns presented as having been laid to rest. The data revealed a significant increase (P<0.001) in comfort rating after safety concerns were laid to rest, although the mean rating was still 3.275. (1.14 MB TIF) [file pone.0014322.s002.tif]

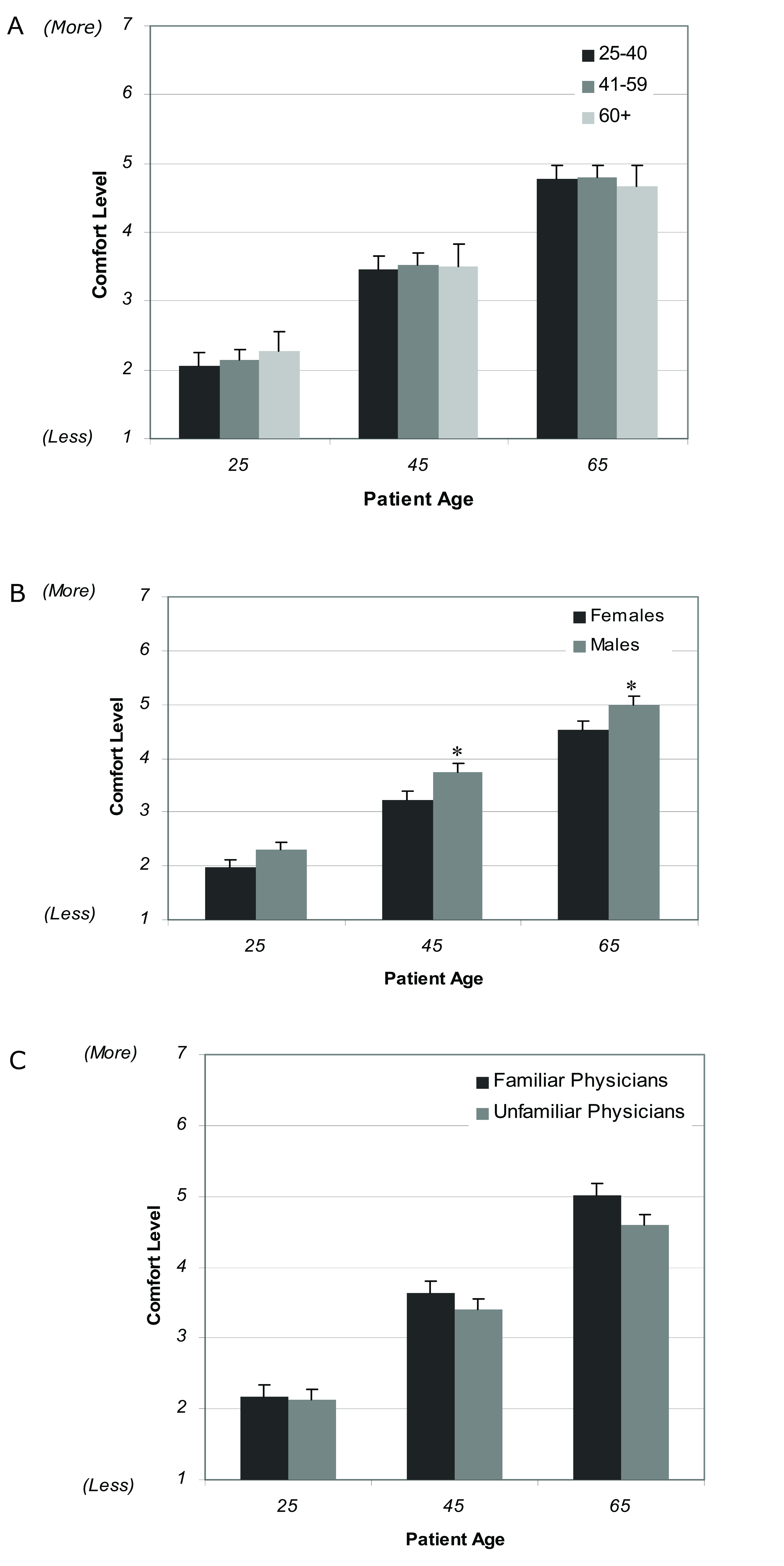

Supplement: Figure S3 — Stratified Analysis of Physicians' Comfort Rating with Prescribing Cognitive Enhancers to Patients. Mean physician comfort rating with prescribing the hypothetical cognitive enhancer to patients of differing ages stratified by physician age, sex, and familiarity with cognitive enhancers. (A) There was no significant difference between physicians in different age groups (25–40; 41–59; 60+), P>0.05. (B) Male physicians were significantly more comfortable with prescribing the cognitive enhancer to 45- and 65-year-old patients (P<0.05) compared to the female phyicians. (C) There was no significant difference (P>0.05) in comfort level between physicians who rated themselves as being “familiar” or “unfamiliar” with cognitive enhancement in healthy persons. (2.42 MB TIF) [file pone.0014322.s003.tif]

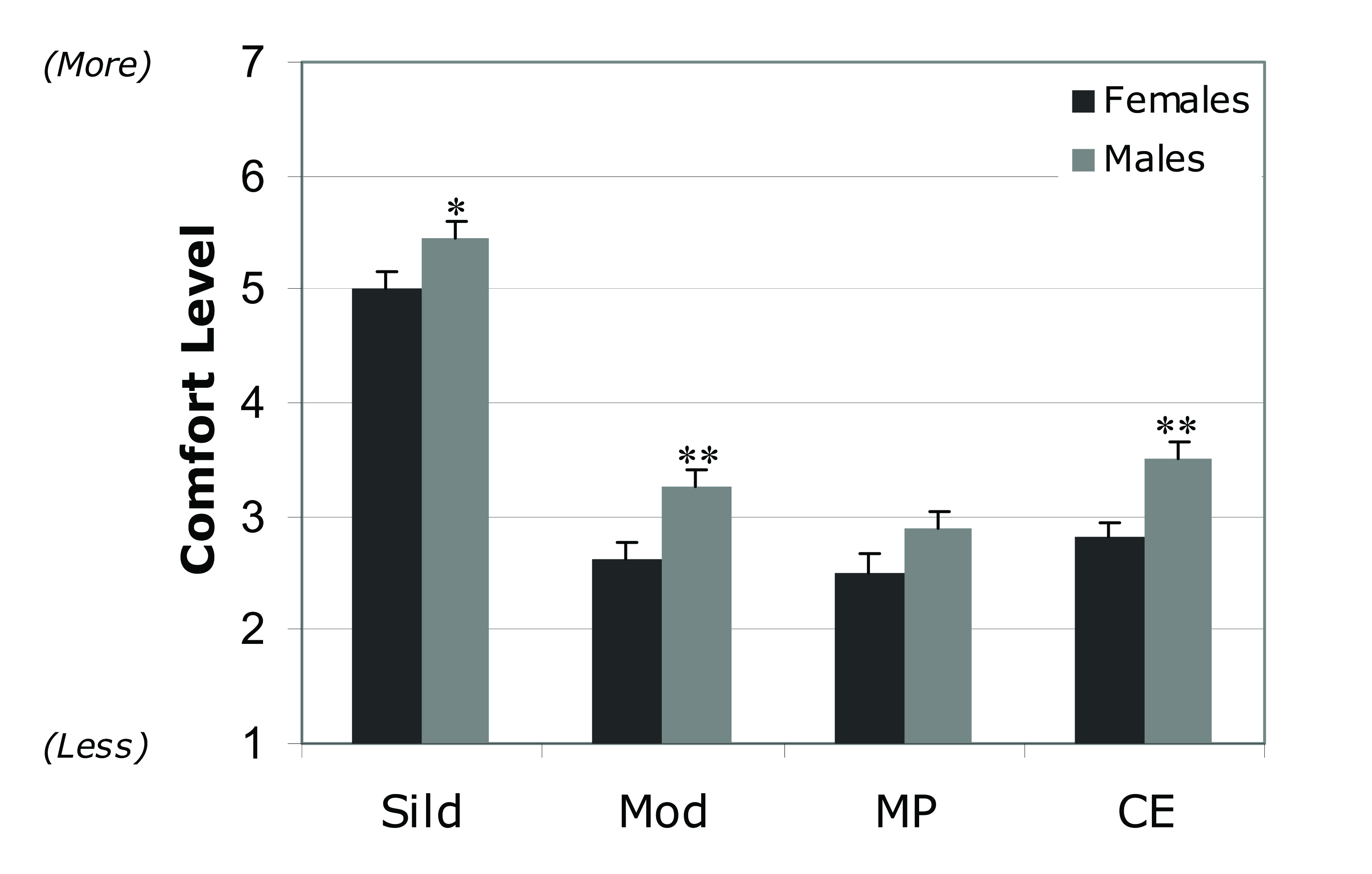

Supplement: Figure S4 — Male and Female Physicians' Comfort Rating with Prescribing Sildenafil, Methylphenidate, Modafinil, and the Cognitive Enhancer. Male physicians reported being significantly more comfortable prescribing sildenafil (P<0.05); modafinil (P<0.005); and the hypothetical cognitive enhancer (P<0.005), when compared with female physicians. (1.16 MB TIF) [file pone.0014322.s004.tif]
