# Supplementary material for: Physician Attitudes towards Pharmacological Cognitive Enhancement: Safety Concerns Are Paramount
Source: PLoS One. 2010 Dec 14;5(12):e14322. doi: 10.1371/journal.pone.0014322 (PMC3001858; doi:10.1371/journal.pone.0014322)
Supplement: Table S1 — Selected Comments on Physician Views on Prescribing Cognitive Enhancers. (0.03 MB DOC) [file pone.0014322.s005.doc]

| **Theme** | **Comments** |
| --- | --- |
| Safety | - It would take a generation to have good long-term safety data. By then, all currently practicing MDs would be near retirement. I would not want to be a guinea pig nor my patients. If 20 years from now we have good safety data then I have less objection, but it still seems unethical. - I’d be very weary about taking or prescribing any medication especially if it was not treating any disease unless I were completely convinced that it has been adequately tested for SEs AND already used/tried for quite a bit of time(years) with good results and with no reports of any ill effects. - I am hesitant to use any medication in an otherwise healthy person. I’m never convinced there will be a med that will do this without short term or long-term side effects. I am also concerned about the cost of a new medication and the difference in the availability of the medication to more affluent people who have insurance vs. those who cannot afford a new brand name, non-preferred higher cost med. - We have had many new drugs deemed "safe" that proved to be otherwise when used widely. A role of the family physicians is to articulate this for ALL new drugs. Any apprehensions I have about this class are no different than any new class. Family docs take statements such as “safe, no side effects” with a grain of salt for ALL new drugs. This has influenced my answers. - The amount of benefit weighed against the amount of risk combined with the cost of drug development makes these types of drugs seemingly ridiculous in my mind. So much money invested in medications that are unlikely to alter performance significantly, and most likely WILL have some adverse effects. - I am very suspicious of such claims until such a drug has been on the market for at least a decade or two!!!!!!!!!!!!!!!!!!!!!!! - NOTHING HAS BEEN MENTIONED ABOUT SAFETY AND DOUBLE BLINDED STUDIES OF AT LEAST 100000 INDIVIDUALS. |
| Physician discomfort with enhancement | - I don't believe in prescribing medications to someone who is otherwise healthy to enhance his cognition - Though declared as "safe", I don't think pharmaceutical cognitive enhancers are ethically right. - Uncomfortable with the idea |
| Beyond the proper goals of medicine | - We have been 'raised' in a disease oriented medical system. The use of a cognitive enhancer to counter normal aging is beyond the scope of my training. Preventive care, anticipatory guidance, and treatment of disease are within my perception of my role. |
