# Supplementary material for: Physician Attitudes towards Pharmacological Cognitive Enhancement: Safety Concerns Are Paramount
Source: PLoS One. 2010 Dec 14;5(12):e14322. doi: 10.1371/journal.pone.0014322 (PMC3001858; doi:10.1371/journal.pone.0014322)
Supplement: Table S2 — Physician Reasons for Probing or not Probing Cognitive Health in Patients. Table S2 shows the percentage of physicians that selected individual reasons from the list we offered as to why they probe or do not probe cognitive health in patients of different age groups during routine visits. Respondents were able to select as many of the reasons as they felt was applicable. (0.04 MB DOC) [file pone.0014322.s006.doc]

| ***Reasons for Probing*** | ***25-40*** | ***41-59*** | ***60+*** |
| --- | --- | --- | --- |
| Important part of overall health and wellness | 73% | 77% | 78% |
| Age-appropriate | 36% | 55% | 84% |
| Patient showing symptoms of cognitive deficits | 52% | 61% | 75% |
| Patient complaining of cognitive deficits | 61% | 76% | 77% |
| To create awareness about normal brain aging | 24% | 43% | 45% |
| Knowledge of patient’s family history | 48% | 43% | 41% |
| Familiarity with patient | 30% | 41% | 38% |
| Patient’s socio-economic status | 21% | 16% | 10% |
| ***Reasons for not Probing*** | ***25-40*** | ***41-59*** | ***60+*** |
| Not a serious medical condition | 12% | 9% | 4% |
| Not age-appropriate | 52% | 27% | 2% |
| Patient not showing symptoms of cognitive deficits | 81% | 85% | 73% |
| Patient not complaining of cognitive deficits | 79% | 86% | 78% |
| To avoid embarrassing the patient | 3% | 3% | 4% |
| To avoid creating anxiety | 12% | 14% | 4% |
| Knowledge of patient’s family history | 12% | 14% | 9% |
| Inadequate familiarity with patient | 8% | 8% | 7% |
| Patient’s socio-economic status | 4% | 3% | 0% |
| Time | 51% | 50% | 58% |
