# Supplementary material for: Physician Attitudes towards Pharmacological Cognitive Enhancement: Safety Concerns Are Paramount
Source: PLoS One. 2010 Dec 14;5(12):e14322. doi: 10.1371/journal.pone.0014322 (PMC3001858; doi:10.1371/journal.pone.0014322)
Supplement: Table S3 — Comments on Prescribing Cognitive Enhancers to a 25-year-old, Assuming Long-term Favorable Safety Data. Physicians were asked to freely respond on the question of prescribing the hypothetical cognitive enhancer to a 25-year-old patient assuming all the safety concerns they previously had have been laid to rest with long-term convincing data. Their comments were grouped into themes using the conceptual analysis method. (0.03 MB DOC) [file pone.0014322.s007.doc]

| ***Themes*** | ***Percentage of Comments*** |
| --- | --- |
| Age-appropriateness | 20% |
| Treatment-focused Physician | 18% |
| Physician discomfort with enhancement | 16% |
| Distrust of pharmaceutical safety data | 16% |
| Cheating/fairness concerns | 14% |
| Comfortable with safety clarifications | 14% |
| Still need more information to prescribe | 8% |
| Distributive Justice | 4% |
| Availability of Non-pharmacological Alternatives | 4% |
| Efficacy concerns | 2% |
