# Supplementary material for: Physician Attitudes towards Pharmacological Cognitive Enhancement: Safety Concerns Are Paramount
Source: PLoS One. 2010 Dec 14;5(12):e14322. doi: 10.1371/journal.pone.0014322 (PMC3001858; doi:10.1371/journal.pone.0014322)
Supplement: Table S4 — Physician Comments on being more Comfortable Prescribing Sildenafil Compared to the Other Three Drugs. Physicians were asked to freely respond on why they feel the data showed that the majority of the respondents were significantly more comfortable prescribing sildenafil compared to the other 3 drugs. Their comments were grouped into themes using the conceptual analysis method. (0.03 MB DOC) [file pone.0014322.s008.doc]

| ***Themes*** | ***Percentage of Comments*** |
| --- | --- |
| Increased familiarity | 27% |
| Better safety profile | 24% |
| Concerns about addictive/abuse potential | 16% |
| Neural vs. somatic targets | 14% |
| Ability to better determine “success” of medication | 14% |
| Concerns about how frequently drug needs to be used | 8% |
| Sildenafil considered a treatment and not an enhancement | 8% |
| Proven efficacy | 5% |
| Increased advertisement | 5% |
| Authenticity concerns about the other 3 drugs | 5% |
